# Genome-Wide Assessment of Population Structure and Genetic Diversity of the Global Finger Millet Germplasm Panel Conserved at the ICRISAT Genebank

**DOI:** 10.3389/fpls.2021.692463

**Published:** 2021-08-20

**Authors:** C. Backiyalakshmi, Mani Vetriventhan, Santosh Deshpande, C. Babu, V. Allan, D. Naresh, Rajeev Gupta, Vania C. R. Azevedo

**Affiliations:** ^1^Centre for Plant Breeding and Genetics, Tamil Nadu Agricultural University (TNAU), Coimbatore, India; ^2^International Crops Research Institute for the Semi-Arid Tropics (ICRISAT), Hyderabad, India

**Keywords:** finger millet, germplasm, genetic diversity, population structure, DArTseq, Analysis of molecular variance (AMOVA)

## Abstract

Finger millet [*Eleusine coracana* (L.) Gaertn.] is an important climate-resilient nutrient-dense crop grown as a staple food grain in Asia and Africa. Utilizing the full potential of the crop mainly depends on an in-depth exploration of the vast diversity in its germplasm. In this study, the global finger millet germplasm diversity panel of 314 accessions was genotyped, using the DArTseq approach to assess genetic diversity and population structure. We obtained 33,884 high-quality single nucleotide polymorphism (SNP) markers on 306 accessions after filtering. Finger millet germplasm showed considerable genetic diversity, and the mean polymorphic information content, gene diversity, and Shannon Index were 0.110, 0.114, and 0.194, respectively. The average genetic distance of the entire set was 0.301 (range 0.040 – 0.450). The accessions of the race *elongata* (0.326) showed the highest average genetic distance, and the least was in the race *plana* (0.275); and higher genetic divergence was observed between *elongata* and *vulgaris* (0.320), while the least was between *compacta* and *plana* (0.281). An average, landrace accessions had higher gene diversity (0.144) and genetic distance (0.299) than the breeding lines (0.117 and 0.267, respectively). A similar average gene diversity was observed in the accessions of Asia (0.132) and Africa (0.129), but Asia had slightly higher genetic distance (0.286) than African accessions (0.276), and the distance between these two regions was 0.327. This was also confirmed by a model-based STRUCTURE analysis, genetic distance-based clustering, and principal coordinate analysis, which revealed two major populations representing Asia and Africa. Analysis of molecular variance suggests that the significant population differentiation was mainly due to within individuals between regions or between populations while races had a negligible impact on population structure. Finger millet diversity is structured based on a geographical region of origin, while the racial structure made negligible contribution to population structure. The information generated from this study can provide greater insights into the population structure and genetic diversity within and among regions and races, and an understanding of genomic-assisted finger millet improvement.

## Introduction

Finger millet [*Eleusine coracana* (L.)] is an important nutraceutical crop. It is highly adapted to the semiarid tropics and is grown as a staple food crop in Asia and Africa. Globally, it is the sixth most important crop among cereals in terms of production, and it contributes about 12% of the total millet area (Mundada et al., 2020). Its origin dates back to 5,000 years in western Uganda and the Ethiopian highlands. In India, its cultivation can be traced to 3,000 BC in the Western Ghats; thus, India is considered a secondary center of diversity for finger millet (Hilu and DeWet, 1976; Hilu et al., 1979). The crop is highly self-pollinated and allotetraploid (AABB) with chromosome number 2n = 4x = 36. Finger millet is gaining importance and drawing attention globally due to its grain nutrient composition, with high dietary fiber (11–20%), essential amino acids, vitamins, and micronutrients, particularly calcium (1.8–4.9 g/kg), iron (~22–65 mg/kg), zinc (~17–25 mg/kg), protein (6–11%), carbohydrates (65–75%), and other minerals; it is also gluten-free (Chethan and Malleshi, 2007; Upadhyaya et al., 2011; Shobana et al., 2013; Devi et al., 2014; Longvah et al., 2017). This enables it to deliver multiple benefits in terms of health compared with major cereals (Saleh et al., 2013). Finger millet has been identified as one of the “future smart food crops” by FAO (Li and Siddique, 2018) because of its nutrient-dense and climate-resilient features; moreover, it can produce a reasonable yield at a relatively low cost of cultivation (Gupta et al., 2017). Finger millet grains are highly resistant to pest attacks and can be stored for long (Iyengar et al., 1945; Mgonja et al., 2007) and provide nutritional support to countries in the developing world (Mgonja et al., 2007; Gupta et al., 2017).

Germplasm is the basic raw material to drive any crop improvement. Its genetic characterization can lead to exploring the variation in germplasm. The great diversity in finger millet comes from its gene pool, including different races and subraces of cultivated species and nine genus *Eleusine* species (Sood et al., 2016; Mirza and Marla, 2019; Vetriventhan et al., 2020). While a large number of finger millet germplasms have been collected and conserved in repositories worldwide, a small fraction has been exploited for economically important traits. The genebank at the International Crops Research Institute for the Semi-Arid Tropics (ICRISAT), Hyderabad, conserves over 7,500 finger millet germplasms (http://genebank.icrisat.org). To enhance the use of diversity in crop improvement, core and mini-core collections were established in finger millet (Upadhyaya et al., 2006, 2010). For this study, we formed a large germplasm diversity representative subset of 314 accessions, originating from 23 countries. The subset included all the mini-core accessions, trait-specific sources identified from the core collection, selections by breeders, and recently assembled germplasm and elite breeding lines. Assessing the diversity and structure of this subset is important for its effective utilization in genomic-assisted crop improvement.

Although nutritional potential and climate resilient features of finger millet are being documented at the global level, it continues to be an orphan crop, lacking genomic information that can be used in crop improvement (Sood et al., 2016). The recent publication of the genome of finger millet (Hittalmani et al., 2017; Hatakeyama et al., 2018) has opened up opportunities to expand genome-level knowledge. Characterization of finger millet germplasm has been assessed, using morphological, and several molecular markers, such as RAPD (Fakrudin et al., 2004; Babu et al., 2007; Das and Misra, 2010; Gupta et al., 2010; Kumari and Pande, 2010; Panwar et al., 2010b; Singh and Kumar, 2010; Ramakrishnan et al., 2016b), ISSR (Salimath et al., 1995; Gupta et al., 2010), SSR (Srinivasachary et al., 2007; Dida et al., 2008; Panwar et al., 2010a,b; Bharathi, 2011; Kumar et al., 2012; Arya et al., 2013; Babu et al., 2014a, 2017; Kalyana Babu et al., 2014; Gimode et al., 2016; Pandian et al., 2018), and EST-SSR (Arya et al., 2009; Naga et al., 2012; Babu et al., 2014b; Bwalya et al., 2020). A few SNP-based studies too were attempted (Gimode et al., 2016; Kumar et al., 2016) to conduct GWAS studies for major agronomic and nutritional traits (Sharma et al., 2018; Puranik et al., 2020; Tiwari et al., 2020). Recent developments in next-generation sequencing have enabled the rapid genotyping of a larger number of germplasms, with a high number of marker loci at low cost. Recently, DArTseq-based SNPs have been used in many crops, especially in orphan crops and tree species where only limited genomic resources are available (Evanno et al., 2005; Kilian et al., 2012; Edet et al., 2018; O'Connor et al., 2019). DArTseq combines DArT with genotyping-by-sequencing technology, with the advantages of better genome coverage, high reproducible markers, and low cost of high throughput genotyping (Allan et al., 2020). This study aims to (i) assess the genetic diversity and population structure of a finger millet global diversity panel, (ii) assess the relationship among races, regions, landraces, and breeding lines, and (iii) identify the most diverse accessions. The information generated from this study can support understanding of the population structure and genetic diversity of finger millet germplasm, utilize the novel diversity to broaden the genetic base, and also accelerate genomics-assisted finger millet improvement.

## Materials and Methods

### Plant Materials

**Figure 1:**
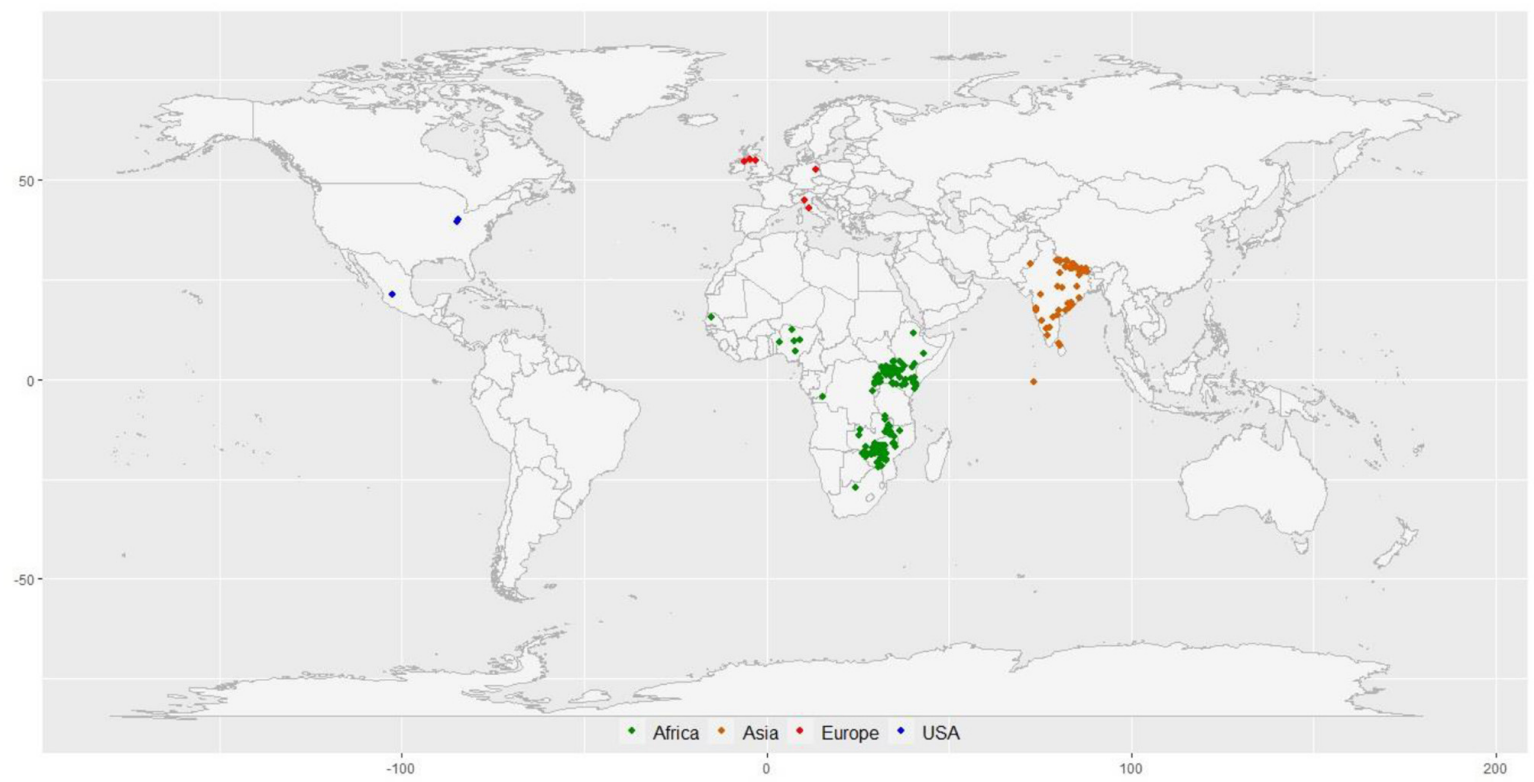
The geographical distribution of 297 finger millet accessions with known geo-coordinates distributed in 23 countries around the world.

A diversity panel of 314 finger millet accessions originating from 23 different countries was constituted for this study (Figure 1; Supplementary Table 1). These accessions represent four geographical regions in the world: Africa (160), Asia (136), Europe (6), North America (3), and unidentified origin (9). The panel represents all the races and subraces of finger millet: *vulgaris* (202 accessions), *plana* (48 accessions), *elongata* (31 accessions), *compacta* (28 accessions), and some unclassified (5 accessions). Each race was further grouped into subraces, except *compacta* that have no subrace. The diversity panel consists of both landraces (264 accessions) and breeding material (50 accessions) (Supplementary Table 1).

### DNA Extraction and DArT Sequencing

A total of 314 samples (including 4 control varieties, GPU 26, MR 6, KMR 204, and VL 149) were used for genotyping. Eight seeds of each accession were randomly chosen to constitute a sample for DNA extraction and sequencing. The seed samples from the 2018 rainy season harvested material were cleaned properly to avoid contamination by dust particles. The seeds were then placed in each well of the PCR plate and tightly sealed to avoid contamination. DNA extraction and sequencing were done by DArT private limited (www.diversityarrays.com); details regarding DArT genotyping methods and procedures can be found at http://www.diversityarrays.com/dart-application and in Kilian et al. (2012). The finger millet germplasm used in this study was conserved in the genebank at ICRISAT, Hyderabad, and is available to researchers globally, following the standard material transfer agreement (SMTA).

## Data Analysis

### SNP Filtering

The SNP markers obtained from DArTseq were filtered with a maximum threshold of 95% reproducibility, <20% missing values for markers, and <50% missing values for each accession to obtain high-quality SNP markers, using dartR packages in R software (Gruber et al., 2018).

### Genetic Diversity and Structure Analysis

Locus-based diversity estimates, such as minor allele frequency (MAF), polymorphism information content (Botstein et al., 1980), expected heterozygosity [also known as “gene diversity” (GD)] (Nei, 1973), and Shannon information index, were calculated. The genetic distance matrix was calculated, following the modified Roger's distance (MRD) method (Wright, 1978), and a dendrogram was constructed, using the ward. D2 agglomerative hierarchical clustering method (Murtagh and Legendre, 2014). Principal coordinates analysis (PCoA) was performed, using the distance matrix obtained from MRD. The analysis was performed by custom-scripted codes in R program v.3.6.0 using “*Adegenet*” (Jombart, 2008), “*ade4*” (Dray et al., 2007), and “*cluster*” (Maechler et al., 2019) packages. Population structure was assessed, using STRUCTURE v.2.3.4 software (Pritchard et al., 2000), using an admixture model, with a K-value ranging from 1 to 8, with three independent runs. Burn-in time and Markov Chain Monte Carlo (MCMC) were set up to 10,000/50,000 iterations for each run. The optimal number of genetic groups (K) was determined by STRUCTURE HARVESTER (Earl and VonHoldt, 2012), based on the rate of change in the log probability of data between successive K-values (Evanno et al., 2005).

### Population Differentiation and Genetic Diversity Indices

AMOVA was computed to ascertain the level of genetic differentiation within and among structure-defined populations, regions, races as well as a biological status, using 999 permutations, using R v.3.6.0 package “*poppr*” (Kamvar et al., 2014). Besides, indices that explain the diversity in the population were calculated. Genetic indices, such as Shannon's Index (*I*) and gene diversity (*He*), were computed for regions, races, and biological status using R v.3.6.0 package “*poppr*” (Kamvar et al., 2014).

## Results

### SNP Filtering

A total of 46,336 DArTseq-based SNP markers were generated, and 33,884 polymorphic SNP markers were retained after filtering. Of the 314 finger millet accessions (310 accessions in the diversity panel and four controls), eight accessions (IE 2030, IE 2799, IE 2825, IE 3157, IE 3475, IE 3788, IE 6314, and VL 149) were removed for having >50% missing data; so, all the downstream analyses were carried out only on 306 accessions. The call rate ranged from 80 to 100%, of which 30,869 markers displayed a >85% call rate. Reproducibility ranged from 95 to 100%, and 88.80% of SNP markers showed >97% reproducibility.

### Genetic Diversity Analysis

**Figure 2:**
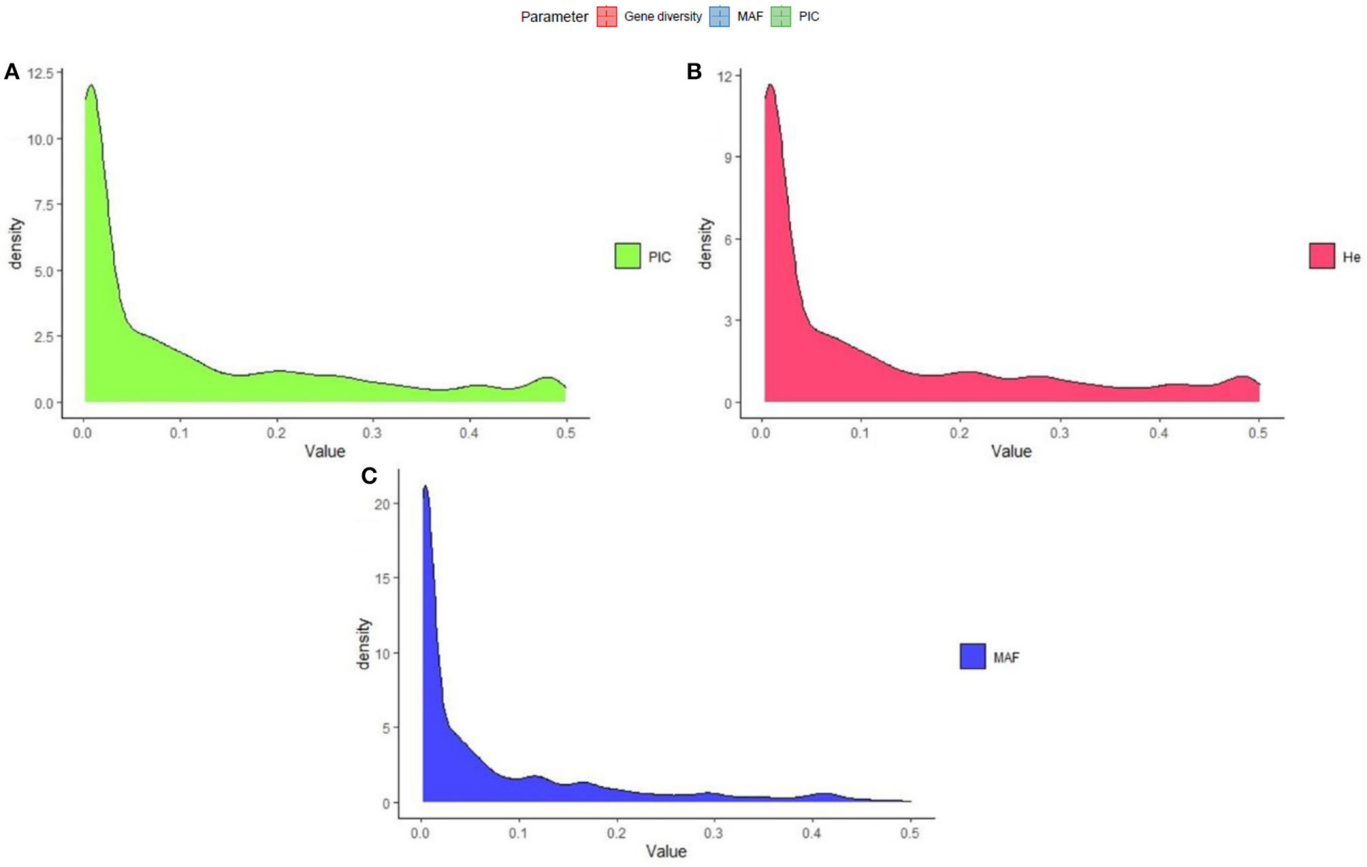
Frequency distribution of quality marker parameters of **(A)** polymorphism information content (PIC), **(B)** gene diversity (He), and **(C)** minor allele frequency (MAF) for DArTseq markers in the finger millet diversity panel.

The polymorphism information content value ranged from 0.003 to 0.500 (maximum value for the biallelic marker) with a mean value of 0.110. The gene diversity for all loci in the entire set of germplasms ranged from 0.001 to 0.50, with an average of 0.114 (Figure 2). The frequency distribution of polymorphism information content (PIC), minor allele frequency (MAF), and gene diversity (He), considering landraces and breeding lines were depicted in the Supplementary Figure 1. It clearly showed the difference in the dynamics of gene diversity and minor allele frequency between landraces and breeding lines. The density graph showed low frequency of rare alleles in breeding lines as compared with landraces for the polymorphic SNPs. Similarly, the gene diversity distribution slope was narrower in breeding lines than in landraces. It shows that landraces hold greater diversity than breeding lines. Mean gene diversity was similar but slightly higher in the Asian (0.132) accessions compared with the African accessions (0.129). Among the races, *vulgaris* and *elongata* had higher gene diversity (0.144), followed by *compacta* (0.142), and the lowest gene diversity was in race *plana* (0.129). Likewise, landraces (0.144) showed greater gene diversity compared with breeding lines (0.117) (Table 1). The African (0.243) and Asian (0.242) accessions had similar Shannon Index values. While among races, *vulgaris* had a higher value (0.268), and *plana* had a low value (0.233). Similarly, landraces (0.273) had a higher Shannon Index than breeding lines (0.204) (Table 1).

**Table 1:** Shannon diversity index and gene diversity for the entire set, regions, races, and biological status of finger millet germplasm.

**Population (number of accessions)**	**Shannon Index (*I*)**	**Gene diversity (*He*)**
**Overall mean**	0.194 ± 0.01	0.114 ± 0.01
**Region**		
Africa (160)	0.243 ± 0.02	0.129 ± 0.01
Asia (136)	0.242 ± 0.03	0.132 ± 0.02
**Races**		
*Compacta* (28)	0.246 ± 0.06	0.142 ± 0.04
*Elongata* (31)	0.241 ± 0.05	0.144 ± 0.03
*Plana* (48)	0.233 ± 0.04	0.129 ± 0.03
*Vulgaris* (202)	0.268 ± 0.02	0.144 ± 0.01
**Biological status**		
Landraces (264)	0.273 ± 0.02	0.144 ± 0.01
Breeding lines (50)	0.204 ± 0.04	0.117 ± 0.03

Genetic distance was assessed by the modified Roger's distance (MRD) method. The genetic distance among individuals ranged from 0.040 to 0.450 with a mean value of 0.301. Among regions, the highest mean genetic distance was found in the Asian accessions (0.286), followed by the African accessions (0.276). The genetic distance of accessions from Europe and North America was 0.247 and 0.217, respectively; however, the sample size in these two regions was low (<10) and was, therefore, ignored for further discussion. Among races, *elongata* had the highest genetic distance (0.326), followed by *vulgaris* (0.299) and *compacta* (0.280), and the lowest was in race *plana* (0.275). The average genetic diversity among landraces was 0.299, while breeding lines had a value of 0.267 (**Table 4**).

The genetic distance between the populations (region, race, and biological status) was measured by MRD and the pairwise estimates of *Fst*. Pairwise *Fst* estimates between Asia and Africa were significant (0.198), which indicates the presence of genetic differentiation between the geographical origin, and the genetic distance between the African and Asian was 0.327. Among races (Table 2), pairwise *Fst* estimate values were near zero, indicating no defined population among races. Between races, *vulgaris* and *elongata* had the greatest genetic distance (0.320), while *compacta* and *plana* had the lowest (0.281). The distance between *vulgaris* and *compacta* (0.294) and *vulgaris* and *plana* (0.302) was low compared with that between *elongata* and *compacta* (0.308) and *elongata* and *plana* (0.310). Significant genetic divergence and *Fst* were observed between landraces and breeding lines (0.313 and 0.136), respectively (Table 2).

**Table 2:** Pairwise estimates of modified Roger's distance (MRD) (diagonal and upper diagonal) and *Fst* (lower diagonal) among the regions, races, and biological status of finger millet germplasm.

**Population**	**MRD and** ***Fst***			
**Region**	**Africa**	**Asia**		
Africa	0.276	0.327		
Asia	0.198^*^	0.286		
**Races**	***Vulgaris***	***Compacta***	***Plana***	***Elongata***
*Vulgaris*	0.299	0.294	0.302	0.320
*Compacta*	0.024^*^	0.280	0.281	0.308
*Plana*	0.068^*^	0.022	0.275	0.310
*Elongata*	0.038^*^	0.027^*^	0.044^*^	0.326
**Biological status**	**Landraces**	**Breeding lines**		
Landraces	0.299	0.313		
Breeding status	0.136^*^	0.267		

* *Significant at p = 0.05 level*.

### Most Diverse Accessions

Considering the relatedness of the accessions, the most diverse individual accessions and pairs of accessions were identified (Table 3). Accession IE 6095, originating in Asia (Nepal), had the highest genetic distance with IE 2606 (0.450), originating in Africa (Malawi). IE 6095 also showed higher genetic distance with four accessions originating in Africa—IE 3399 (0.449), IE 8599 (0.449), IE 2869 (0.442), and IE 5291 (0.440). The 10 most diverse accessions were identified, of which seven (IE 5903, IE 6095, IE 6221, IE 6074, IE 6165, IE 5957, and IE 6059) were from Asia and three (IE 2869, IE 2645, and IE 2780) were from Africa. Accession IE 5903, originating in Asia, was the most distant from other accessions with an average of 0.369. Among African accessions, IE 2869 was the most divergent, with a mean of 0.368.

**Table 3:** Genetically distant individuals and pairwise accessions identified using the modified Roger's distance method.

**Accessions**	**Region**	**Country**	**Average Genetic distance from other accessions**
IE 5903	Asia	Nepal	0.369
IE 2869	Africa	Zambia	0.368
IE 6095	Asia	Nepal	0.366
IE 2645	Africa	Malawi	0.360
IE 6221	Asia	Nepal	0.355
IE 6074	Asia	Nepal	0.355
IE 6165	Asia	Nepal	0.353
IE 5957	Asia	Nepal	0.353
IE 6059	Asia	Nepal	0.351
IE 2780	Africa	Malawi	0.351
**Diverse pair of accessions**			**Genetic distance**
IE 6095 Asia (Nepal) and IE 2606 Africa (Malawi)			0.450
IE 6095 Asia (Nepal) and IE 3399 Africa (Zimbabwe)			0.449
IE 6095 Asia (Nepal) and IE 8599 Africa (Kenya)			0.449
IE 5903 Asia (Nepal) and IE 8599 Africa (Kenya)			0.447
IE 5903 Asia (Nepal) and IE 3399 Africa (Zimbabwe)			0.446
IE 5903 Asia (Nepal) and IE 2606 Africa (Malawi)			0.444
IE 6165 Asia (Nepal) and IE 2606 Africa (Malawi)			0.442
IE 6095 Asia (Nepal) and IE 2869 Africa (Zambia)			0.442
IE 6221 Asia (Nepal) and IE 2606 Africa (Malawi)			0.441
IE 6221 Asia (Nepal) and IE 8599 Africa (Kenya)			0.441
IE 6165 Asia (Nepal) and IE 8599 Africa (Kenya)			0.441
IE 5903 Asia (Nepal) and IE 2645 Africa (Malawi)			0.440
IE 6095 Asia (Nepal) and IE 5291 Africa (Zimbabwe)			0.440
IE 6221 Asia (Nepal) and IE 3399 Africa (Zimbabwe)			0.439
IE 3130 Asia (India) and IE 2780 Africa (Malawi)			0.439

### Population Structure

**Figure 3:**
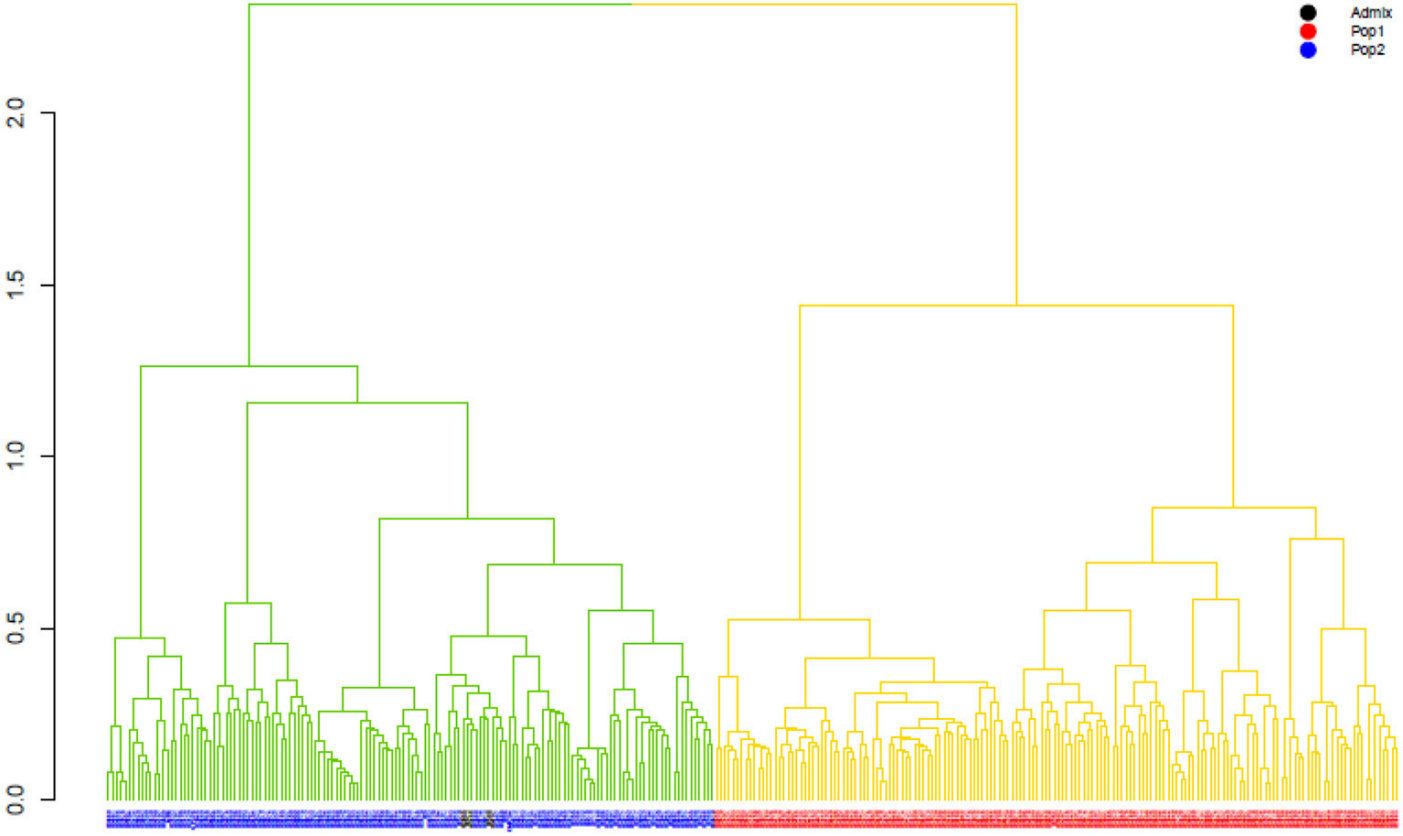
The ward.D2 agglomerative hierarchical clustering analysis of finger millet diversity panel, using DArTseq-based modified Roger's distance. Note: colored branches: cluster I – Asia (green); cluster II – Africa (yellow); colored accessions-subclustering based on STRUCTURE results.

**Figure 4:**
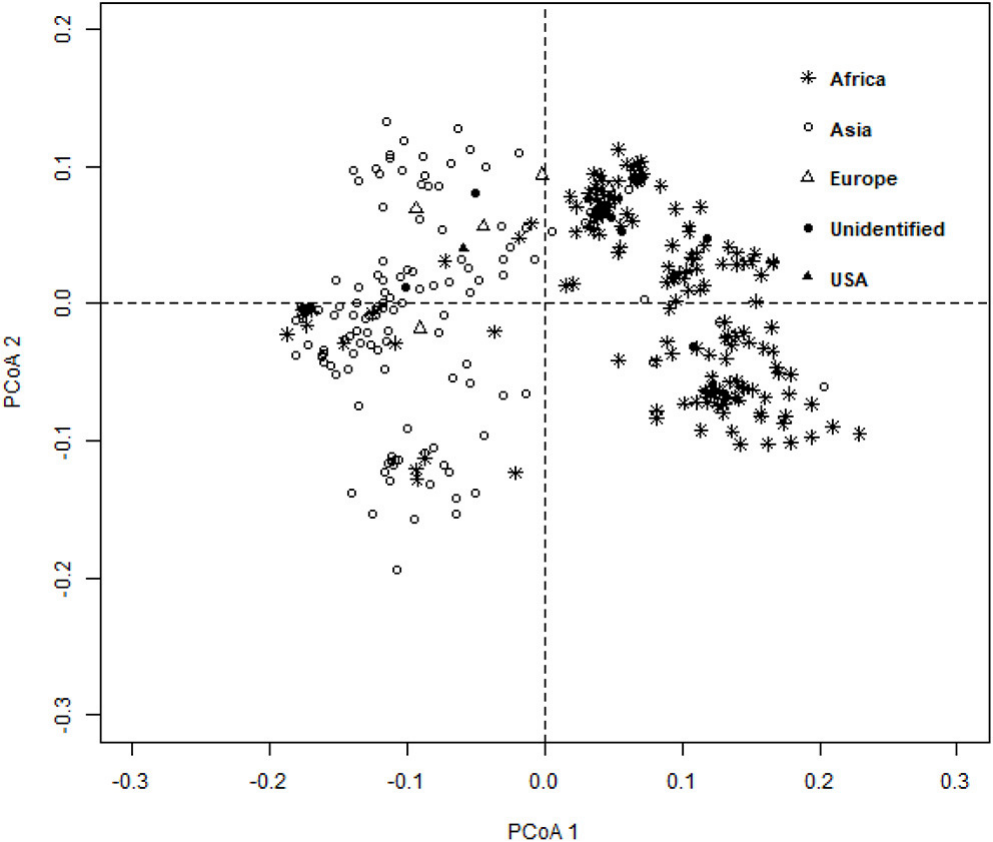
Principal coordinates analysis (PCoA) based on the modified Roger's distance of finger millet diversity panel.

The ward.D2 agglomerative hierarchical clustering method, representing the relationship based on MRD, showed that 306 finger millet accessions were split into two distinct clusters (cluster I – Asia, and cluster II – Africa) based on geographical origin (Table 4; Supplementary Table 2). Cluster I consisted of 144 individuals, the majority from Asia (125), and cluster II comprised 162 individuals, of which 142 accessions were from Africa. Accessions originating in Europe and North America were present in both clusters. Accessions with an unidentified origin were mostly grouped in cluster II (seven accessions) than in cluster I (two accessions) (Figure 3). Principal coordinates analysis (PCoA) revealed the two distinct clusters that were based on geographical origin, suggesting that distinct genetic structure exist between African and Asian finger millet accessions. The results shown in Figure 4 were in concordance with the clustering pattern of the dendrogram, and the first two principal coordinates account for 32.6% (22.3 and 10.3%) of the total observed variation.

**Table 4:** Dendrogram results and mean genetic distance of geographical regions, races, and biological status of the finger millet diversity panel.

		**Cluster I(144 genotypes)**	**Cluster II** **(162 genotypes)**	**Average distance**
Region	Africa	13	142	0.276
	Asia	125	8	0.286
	Europe	3	3	0.247
	North America	1	2	0.217
	Unidentified	2	7	0.271
Race	*Compacta*	9	18	0.280
	*Elongata*	11	18	0.326
	*Plana*	5	43	0.275
	*Vulgaris*	115	82	0.299
	Unclassified	4	1	0.291
Biological status	Landraces	97	160	0.299
	Breeding lines	47	2	0.267
Average distance	Within cluster	0.263	0.282	
	Between cluster	0.332		
	Overall range	0.040–0.450		
	Overall mean	0.301		

**Figure 5:**
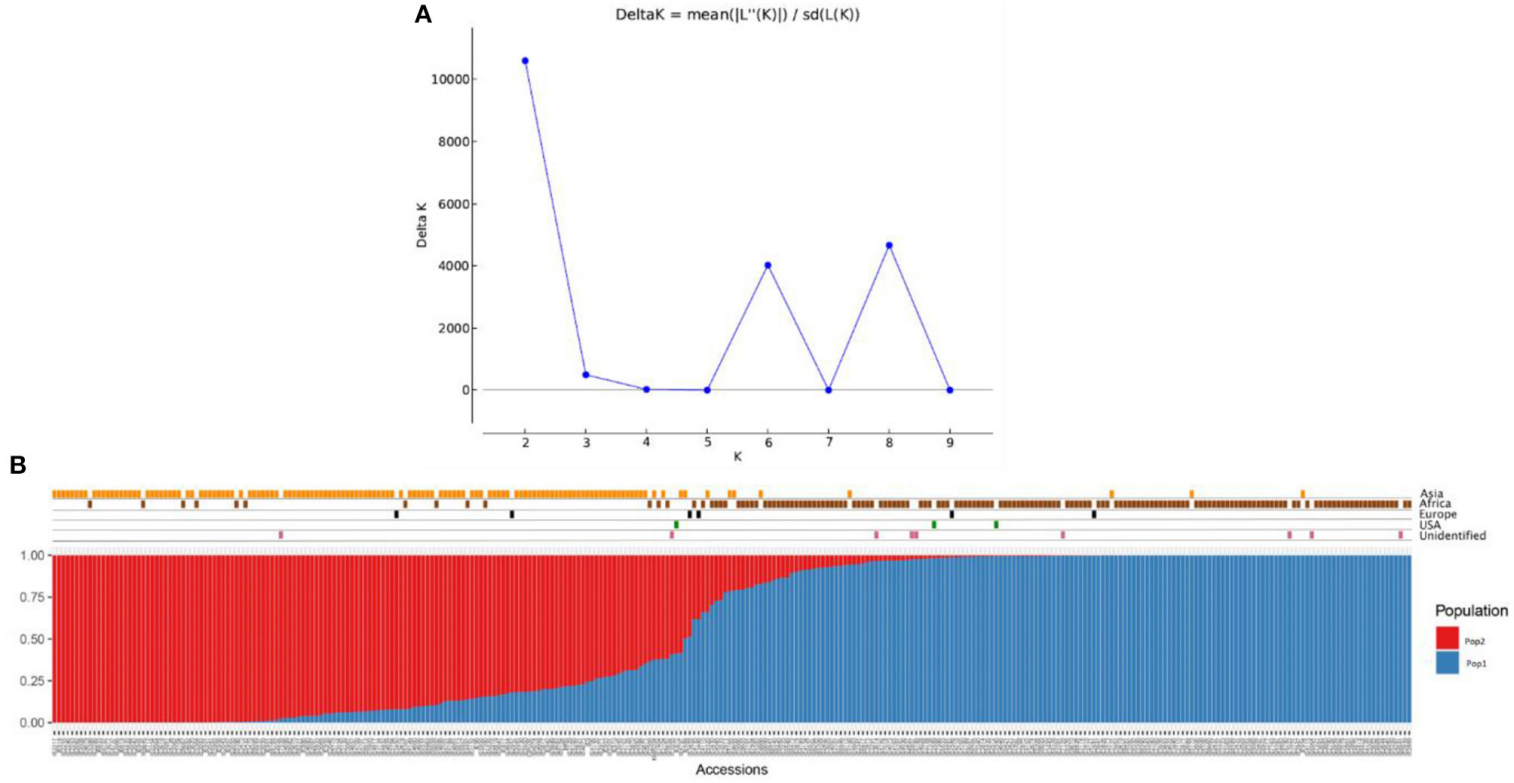
Population structure analysis of the finger millet diversity panel based on DArTseq markers: **(A)** Delta K- based on the rate of change in [LnP (D)] between successive K-values of populations and **(B)** estimated population structure at *K* = 2 on geographical regions.

The population structure among the 306 finger millet accessions was assessed with STRUCTURE v.2.3.4, and the results revealed the existence of two major populations (pop1 and pop2) according to a geographical region of origin (K = 2) (Figures 5A,B). The accessions in pop1 were mostly from Africa (87%) and those in pop2 mostly from Asia (88%). The accessions in pop1 were 142 from Africa, 8 from Asia, 3 from Europe, 2 from North America, and 7 of unidentified origin. Pop2 accessions consisted of 13 from Africa, 123 from Asia, 2 from Europe, and 1 of unidentified origin. A total of five accessions (Asia-2; Europe-2; North America-1; and Unidentified origin-1) not grouped in any of the two populations were considered admixture lines. Apart from that, the admixture of alleles between two subpopulations does exist, and pop2 had more admixture than pop1. The fixation index (*Fst*) estimated from STRUCTURE results for each of the two subpopulations was 0.548 and 0.622, and the ancestry-inferred cluster proportion of the membership of the samples was 0.644 and 0.356. The gene diversity of the two subpopulations was 0.087 and 0.093 (Table 5).

**Table 5:** Gene diversity and *Fst* of the finger millet diversity panel for the two STRUCTURE -based populations.

**K=2**	**Inferred clusters**	***Fst***	**Gene diversity**	**No. of accessions**	**Regional distribution of accessions**
Pop1	0.644	0.548	0.087	162	Africa (142), Asia (8), Europe (3), North America (2), Unidentified (7)
Pop2	0.356	0.622	0.093	139	Africa (13), Asia (123), Europe (2), Unidentified (1)
Admixture				5	Asia (2), Europe (1), North America (1), Unidentified (1)

In addition to population structure at K = 2, the STRUCTURE results showed two more peaks at K = 6 and K = 8 (Figure 5A). Interestingly, the populations at K = 6 and K = 8 were sub-structured from the major populations at K = 2 (Supplementary Table 3). In K = 6, accessions from Africa and Asia were divided into three groups each results in six populations (K = 6) (Supplementary Figure 2). In the case of K = 8, African and Asian accessions were divided into four and three groups, respectively, of which one population had all admixed individuals (six accessions) (Supplementary Figure 3).

Accessions were not clustered based on racial classification. For instance, 58% of race *vulgaris* was grouped into cluster I, while the remaining 42% was under cluster II (Table 4). Races such as *compacta* and *elongata* were grouped under both clusters (9 and 11 accessions in cluster I and 18 accessions each in cluster II, respectively). The majority of the accessions of race *plana* were grouped under cluster II (89%). Landraces were grouped in both clusters I and II (97 and 160 accessions, respectively). However, most of the breeding lines were grouped in cluster I (94%) since they were mainly developed from an Asian breeding program, particularly in India (Table 4).

### Genetic Differentiation and Diversity Indices

Genetic divergence by analysis of molecular variance (AMOVA) (Table 6) for the STRUCTURE population revealed that 25.17% of the total variation was accounted for regional diversity and 37.90% of the variation within individuals. AMOVA on geographical regions revealed 42.36% variation between individuals within regions, 17.69% variation between regions, and 39.96% within individuals. When AMOVA analysis was assessed between races, most of the genetic variation (52.92%) was split into individuals within the race, while 4.60% reflected among races. AMOVA performed for biological status indicated that 13.24% of the total genetic variance was attributable to biological status diversity and 47.59% of the variation between individuals within a biological status.

**Table 6:** Analysis of molecular variance (AMOVA), using DArTseq markers among STRUCTURE populations, region, races, and biological status of the finger millet diversity panel.

**Groups**	**Partitioning**	**Df**	**Mean Sq**.	**Sigma**	**Percentage of variation**	***P*** **-value**
STRUCTURE	Between populations	1	211019.00	683.22	25.17	0.001
defined population	Between individuals within a population	304	3034.08	1002.55	36.93	0.001
	Within individuals	306	1028.98	1028.98	37.90	0.001
Region	Between region	4	41762.67	455.52	17.69	0.001
	Between individuals within a region	301	3210.61	1090.80	42.36	0.001
	Within individuals	306	1029.00	1029.01	39.96	0.001
Race	Between races	3	12890.28	111.70	4.60	0.001
	Between individuals within a race	301	3594.29	1282.64	52.92	0.001
	Within individuals	306	1029.00	1029.01	42.46	0.001
Biological status	Between biological status	1	60752.64	347.62	13.24	0.001
	Between individuals within a biological status	304	3528.59	1249.79	47.59	0.001
	Within individuals	306	1029.00	1029.01	39.18	0.001

## Discussions

### Finger Millet Diversity Panel

For this study, we established a diversity panel of 314 finger millet accessions, representing all the four races and their sub-races, originating from 23 countries. This set purposefully included the entire mini-core collection (80 accessions) (Upadhyaya et al., 2010) to capture the maximum diversity and all the trait-specific sources identified in the core collection. Besides, new diversity from recently assembled accessions, selection by breeders, and advanced breeding lines or improved cultivars were included. This germplasm set can act as a potential diversity panel for phenotypic and genomic investigations for finger millet improvement.

### Genetic Diversity and Allelic Richness

This study aimed at the genomic characterization of the finger millet diversity panel to understand diversity and population structure. Genotyping, using the DArTseq platform, provided 46,336 SNPs. Quality filters were applied to ensure high-quality markers to lessen the probability of false interpretation of downstream analysis (O'Connor et al., 2019), as genotyping errors occur irrespective of the DNA sequencing method used (Saunders et al., 2007). Locus-based diversity, population diversity, population structure, and genetic differentiation analyses were performed on the filtered high-quality DArTseq-based SNP markers (33,884).

The PIC of a marker is a good index to evaluate genetic diversity and estimate the level of genetic variation expressed by a particular marker. The average PIC value obtained from our study was 0.110 (Figure 2), which was low compared with those obtained (0.150) for 59 cultivated accessions of finger millet, using SNP markers (Gimode et al., 2016), while the same study found a higher PIC value of 0.300 for wild finger millet. Many factors can influence PIC value, such as the breeding behavior of the species, size, and genetic diversity of the collection, the genotyping method, and the genomic location of markers (Singh et al., 2013; Chen et al., 2017). However, finger millet diversity studies have reported moderate to high informative PIC values (0.256 to 0.700), using SSR markers (Panwar et al., 2010a; Bharathi, 2011; Bheema Lingeswara Reddy et al., 2011; Kumar et al., 2012; Babu et al., 2014a; Nirgude et al., 2014; Lee et al., 2017). In this study, the mean gene diversity and Shannon Index were 0.114 and 0.194, respectively, in an entire set (Table 1). Using SSR markers, gene diversity in finger millet has been reported to range from 0.35 to 0.57 (Dida et al., 2008; Babu et al., 2014b; Lee et al., 2017). Similarly, Upadhyaya et al. (2015) observed gene diversity of 0.28 in foxtail millet, using SNP markers; this was higher than finger millet in this study. Since very few diversity studies, using SNP markers, have been attempted in finger millet, more studies are needed for a better understanding of the finger millet germplasm diversity.

According to previous reports, African germplasm is more diverse compared with Asian germplasm (Dida et al., 2008; Panwar et al., 2010b; Bharathi, 2011; Arya et al., 2013; Kalyana Babu et al., 2014; Kumar et al., 2016; Ramakrishnan et al., 2016a; Babu et al., 2017). In this study, we found similar average gene diversity and genetic distance in African (0.129 and 0.276, respectively) and Asian (0.132 and 0.286, respectively) accessions, with slightly higher diversity in Asian than in African accessions. This could be due to the introduction and extensive utilization of African germplasm into the Asian finger millet breeding program, especially for introgression of blast resistance and for new source of diversity, may result in equal or slightly higher diversity in Asian germplasm than in African germplasm. On *Fst* estimates, a value of more than 0.150 was considered as significant to differentiate between two populations (Frankham et al., 2010), as observed between Asia and Africa (Table 2). Moreover, only six accessions from Europe and three from North America were used in this study. Thus, the interpretation of results with fewer accessions from North America and Europe may lead to biased estimates in terms of genetic differentiation. Hence, the results of African and Asian accessions were taken into consideration for further discussion, and significant genetic differentiation was found between Asian and African accessions.

Cultivated finger millet is classified into four races (*elongata, compacta, vulgaris*, and *plana*) based on inflorescence morphology. Among the races, *elongata* had the highest average genetic distance (Table 4). The Shannon Index value indicated that race *vulgaris* was the commonly found ear type in finger millet with a mean genetic distance less than race *elongata*. However, the mean distance of races *compacta* and *plana* was less, leading to the conclusion that both contribute less variability to the germplasm compared with *elongata* and *vulgaris*. However, gene diversity demonstrated that race *vulgaris* and *elongata* hold more diversity than *plana* (Table 1). On *Fst* estimates, all races had values near zero, indicating no genetic differentiation among races but were morphologically distinct from one another in terms of panicle type as the races are primarily classified based on panicle morphology and shape (Hilu and DeWet, 1976) (Table 2). As expected, landraces had the highest gene diversity and genetic distance compared with breeding lines, as greater genetic variability in landraces and lower in breeding lines are indicative of a domestication bottleneck, and high selection pressure during the breeding process led to genetic erosion (Tables 1, 2). There was clear genetic differentiation between landraces and breeding lines (Table 2).

### Population Structure

Geographical origin is a key determinant of population structure in finger millet germplasm (Dida et al., 2008; Kumar et al., 2016). Population structure, PCoA, and MRD clustering patterns of finger millet germplasms were largely consistent with previous classifications (Dida et al., 2008; Kumar et al., 2016; Puranik et al., 2020), which are based on geographical origin (Figures 3, 4, 5B). This demonstrated the genetic differentiation between finger millet accessions originating in Africa and Asia. A strong genetic structure existed in finger millet; thus the selection of accessions based on origin would be more diverse and effective in a finger millet crop improvement program. Cluster I from the dendrogram was in parallel with pop2 (Asia) from STRUCTURE results. Likewise, cluster II was in parallel with pop1 (Africa).

Although our results grouped individuals largely based on a geographical region, there were a few exceptions. For instance, a few Asian accessions were assembled into the African population and *vice versa*, in concurrence with previous reports (Dida et al., 2008; Ramakrishnan et al., 2016a; Sood et al., 2016). Eight Indian accessions (IE 3131, IE 4655, IE 4866, IE 5165, IE 5170, IE 5320, IE 954, and IE 5198) fell under pop1. Accession IE 3131 is Indaf 9, and IE 4655 is GPU 13; both were released cultivars developed through hybridization between Indian and African germplasm; so, its placement under pop1 came as no surprise. IE 954 is a Co 4 variety developed through pure line selection of Palladam ragi landraces in Tamil Nadu, and the reason for its placement in the African population is not known. The remaining accessions were landraces with no information available on them. Likewise, 13 accessions of African origin were structured into the Asian population (pop2), of which five accessions (IE 4497, IE 6396, IE 6645, IE 6652, and IE 6667) were from Zimbabwe and eight accessions (IE 2658, IE 5364, IE 5367, IE 5388, IE 5433, IE 7390, IE 7404, and IE 8602) from Kenya. The possible hypothesis for this could be germplasm exchange between regions and their utilization in breeding programs.

Finger millet originated and was domesticated in about 5000 BC in western Uganda and the Ethiopian highlands and then spread to India in 3000 BC in the Western Ghats of India; thus, India is considered a secondary center of diversity for finger millet (Hilu and DeWet, 1976; Hilu et al., 1979; deWet et al., 1984). In this study, accessions from Europe and North America were clustered in the Asian and African populations, for which there are two possibilities: either low sample size/insufficient samples from these regions to understand diversity, or that they were representing the native regions of the crop (Africa and Asia) and migrated to the respective regions in a breeding program, or through germplasm exchange/trading. Grouping accessions from different regions into a geographical origin show that finger millet germplasm mainly originated from Africa and were later introduced into Asia through a breeding process such as introduction and domestication, and exchange of germplasm led to the spread to other regions in the world. The five admixture accessions, IE 588 from India, IE 3455 from Europe, IE 872 from Mexico, IE4797 from the Maldives, and IE 1055 from an unidentified region, have been identified. The admixture is evidence of the continuous spill over of finger millet germplasm among different countries to date through breeding programs. There was a lower order of structure at K = 6 and K = 8, which signifies that there might be an existence of substructure within the geographical origin of finger millet accessions. However, K = 6 would be more appropriate to explain the population present in the finger millet accessions, while, in K = 8, one population had all admixed individuals (six accessions) (Supplementary Figure 3). Therefore, a structure at K = 8 might not be much informative compared with K = 6. In K = 6, accessions from Africa were structured into three populations (Supplementary Figure 2); of these, two populations consist of accessions from African lowland countries, such as Zimbabwe, Zambia, Malawi, and Tanzania. Accessions in the third population are mainly from African highland countries, such as Ethiopia, Kenya, and Uganda. Similarly, accessions from Asian origin were structured into three populations; of which, two were from India, while Nepalese accessions assembled into a separate population (Supplementary Figure 2). The population structure indicates the chronological order of domestication and introduction of finger millet in Africa and Asia. The African highland race is the most primitive form. Later, the lowland race was evolved from the highland race and subsequently introduced into India, where it formed into distinct gene pool over the period of time (Hilu and DeWet, 1976; deWet et al., 1984). Later, the African highland races were introduced into Nepal. The genetic differentiation and distinct structure between the region of origin (Africa and Asia) in finger millet are well known. In addition, this study gives new insights into the substructure within geographical origin of finger millet, which needs to be explored for crop improvement.

**Figure 6:**
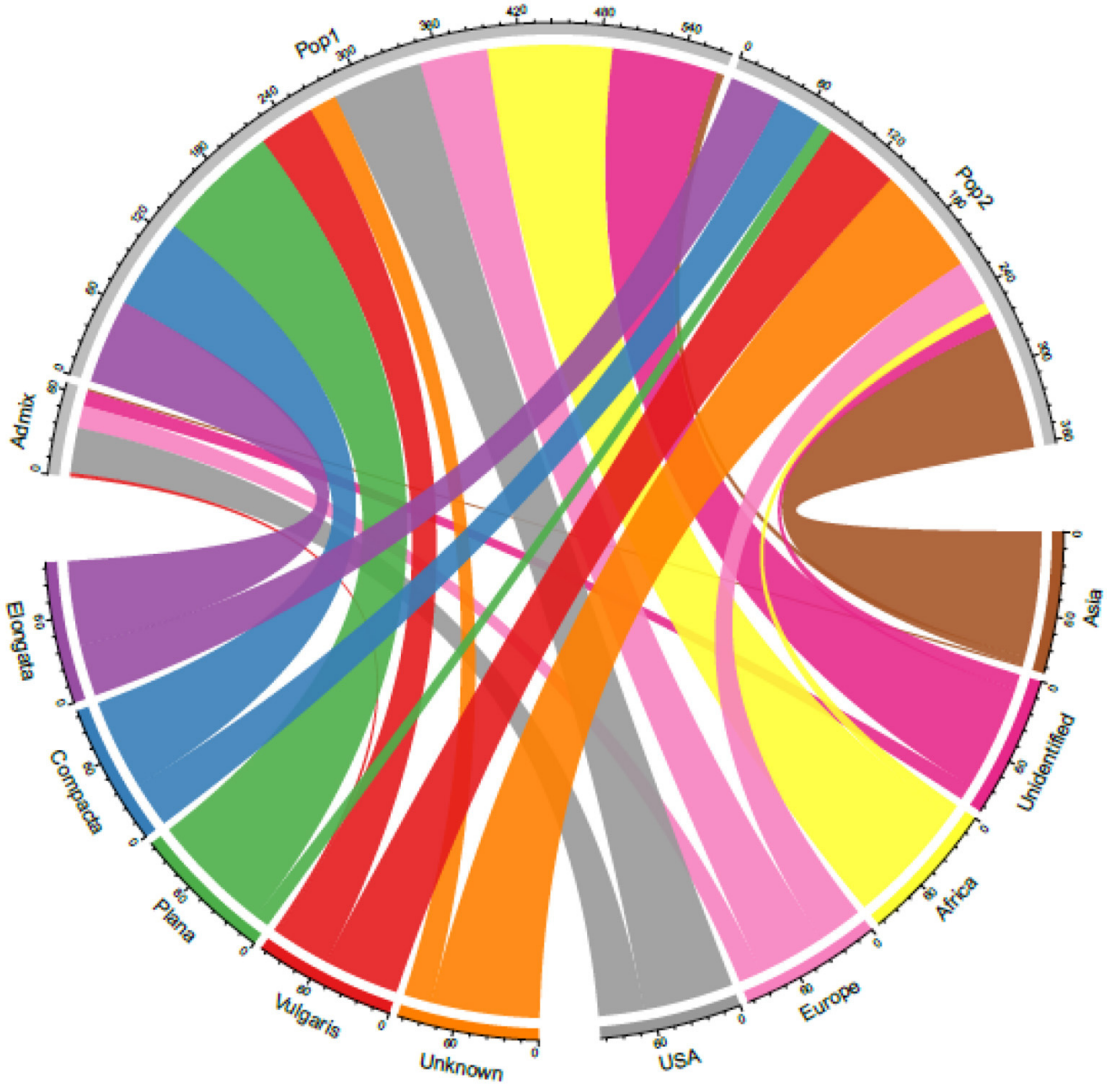
A chord diagram showing the relationship between the STRUCTURE-based populations with the geographical origin and races in the 306 finger millet accessions.

Although finger millet is classified into races and subraces, there are no studies on their impact on diversity and the relationship between races and geographical regions. Studies also have reported the lack of proper clustering among finger millet races (Bharathi, 2011; Kumar et al., 2016) (Table 4). In this study too, the accessions were not structured on the basis of races. This is unlike in foxtail millet, where accessions were structured mainly based on races and regions (Vetriventhan et al., 2014; Upadhyaya et al., 2015). In other small millets, such as proso millet (Vetriventhan et al., 2019), kodo millet (Johnson et al., 2019), and barnyard millet (Wallace et al., 2015), races are not a good indicator of genetic relatedness or to define population structure. The poor grouping among races could be because they were mainly classified based on panicle morphology and shape (deWet et al., 1984) and that the markers used in this study were not sufficient to capture the variation in genomic regions/gene spaces, encoding panicle morphology. On the contrary, the large number of high-quality SNPs does provide a possible capture of additional diversity that is not captured alone with panicle morphology-related gene complexes. This could also indicate that only panicle morphology-based racial differentiation is insufficient to capture the complete diversity in cultivated finger millet. A wide variation in the proportion of races in each cluster was observed, which is similar in the finger miller diversity study by Naik et al. (1993). The variation might be due to the predominance of accessions of race *vulgaris* (64%) compared with other races in the diversity panel and, also, in the entire germplasm of finger millet conserved at the ICRISAT Genebank. Figure 6 explains the relationship between the STRUCTURE population with the geographical regions and races.

Landraces dominate global finger millet germplasm collections conserved in different genebanks compared with improved or breeding lines, which comprise only ~3% of the total collection (Saha et al., 2016; Vetriventhan et al., 2020). The finger millet germplasm collection conserved at the ICRISAT genebank comprises 94% landraces, 3% breeding lines, and 3% wild relatives. The distribution of landraces was maximum in cluster II (62%) compared with cluster I (38%), where 94% of breeding lines were grouped into cluster I (Table 4). This demonstrates that Asian germplasm, in particular Indian accessions, has been subjected to more intense human selection in recent history after domestication. On the other hand, African germplasm remains unexplored and continues to be used mainly as a donor source in finger millet crop improvement programs, particularly in resistance breeding.

### Genetic Differentiation and Diversity Indices

The AMOVA revealed the presence of genetic differentiation among STRUCTURE-derived populations, regions, and biological status (Table 6). The within-population AMOVA analysis explained most of the variance, indicating relatively unrestricted gene flow between regions, races, and biological status. Significant molecular variation was found between the STRUCTURE population and among regions, revealing the existence of genetic differentiation among the geographical regions. The races account for minimal variation in finger millet genetic diversity. For instance, landraces, and breeding lines have an impact on finger millet genetic variation. Altogether, high diversity was observed within populations and between individuals in a population, as reported in the finger millet previous study (Kalyana Babu et al., 2014; Pandian et al., 2018).

### Diverse Accessions

A set of distantly related accessions was identified for use in hybridization and exploitation of hybrid vigor. Hybridization between distantly related individuals tends to yield superior hybrids; the introduction of new allelic combinations has been reported to result in increased heterozygosity (Fasahat et al., 2016). In India, the Indo-African hybridization program was set up to introduce new variability and resistance in finger millet varietal improvement (Dida et al., 2008). It resulted in the release of elite varieties in the country and gained popularity among Indian farmers. The released Indo-African varieties have been used as parents in successive hybridization programs. Consequently, diverse accessions identified from this study could be included in the finger millet varietal improvement program to introduce novel diversity to enable notable advancements in crop productivity. The diverse accessions from Africa include mostly those from African lowlands countries, such as Zimbabwe, Malawi, and Zambia, and Kenya from African highlands. Almost all the top diverse Asian accessions identified were from Nepal. Nevertheless, India is a secondary center of diversity, and improvements within this gene pool will have limited opportunities (Dida et al., 2008). Therefore, the diverse accessions identified from African and Nepalese germplasm need to be explored for use in breeding programs to develop high-yielding cultivars.

## Conclusions

This study provides a detailed understanding of the genetic differentiation between region, races, and biological status, using DArTseq-based SNP markers. The markers differentiated the population structure within the African and Asian regions. Genetic diversity was similar but slightly higher in the Asian accessions compared with the African accessions, probably due to greater integration of alleles from the African accessions through breeding. Finger millet races contribution to diversity was insignificant, and there was less association between geographical region and races; therefore, more attention should go towards the geographical region. The population structure identified in this study will aid in choosing appropriate statistical methods to perform genome-wide association studies (GWAS) and can be used to detect quantitative trait loci (QTLs)/gene in the finger millet population. Insights into finger millet diversity and population structure from this study will help breeders plan their breeding strategy to develop high-yielding cultivars with a broad genetic base for food, nutrition, and environmental security.

## Data Availability Statement

The original contributions presented in the study are included in the article/Supplementary Materials, further inquiries can be directed to the corresponding authors.

## Author Contributions

MV and VCRA contributed to the conception and the design of the study. This work is part of Ph.D. thesis research of CBac. CBab supported student research as chairman. CBac, VA, and MV performed the statistical analysis. CBac and MV wrote the first draft of the manuscript. SD and RG provided the financial and technical support for this study. All the authors contributed to the revision of the manuscript, read, and approved the submitted version.

## Conflict of Interest

The authors declare that the research was conducted in the absence of any commercial or financial relationships that could be construed as a potential conflict of interest.

## Publisher's Note

All claims expressed in this article are solely those of the authors and do not necessarily represent those of their affiliated organizations, or those of the publisher, the editors and the reviewers. Any product that may be evaluated in this article, or claim that may be made by its manufacturer, is not guaranteed or endorsed by the publisher.

## Abbreviations

DArTseq: diversity array technology sequencing
SNP: single nucleotide polymorphism
MAF: minor allele frequency
PIC: polymorphism information content
GD: gene diversity
MRD: modified Roger's distance
PCoA: principal coordinates analysis
MCMC: Markov Chain Monte Carlo
*Fst*: fixation index
AMOVA: analysis of molecular variance
GWAS: genome-wide association studies
QTL: quantitative trait loci.
