# Supplementary material for: Genome-Wide Assessment of Population Structure and Genetic Diversity of the Global Finger Millet Germplasm Panel Conserved at the ICRISAT Genebank
Source: Front Plant Sci. 2021 Aug 20;12:692463. doi: 10.3389/fpls.2021.692463 (PMC8417690; doi:10.3389/fpls.2021.692463)
Supplement: Supplementary file 1 [file Data_Sheet_1.zip › Supplementary Figures.pdf]

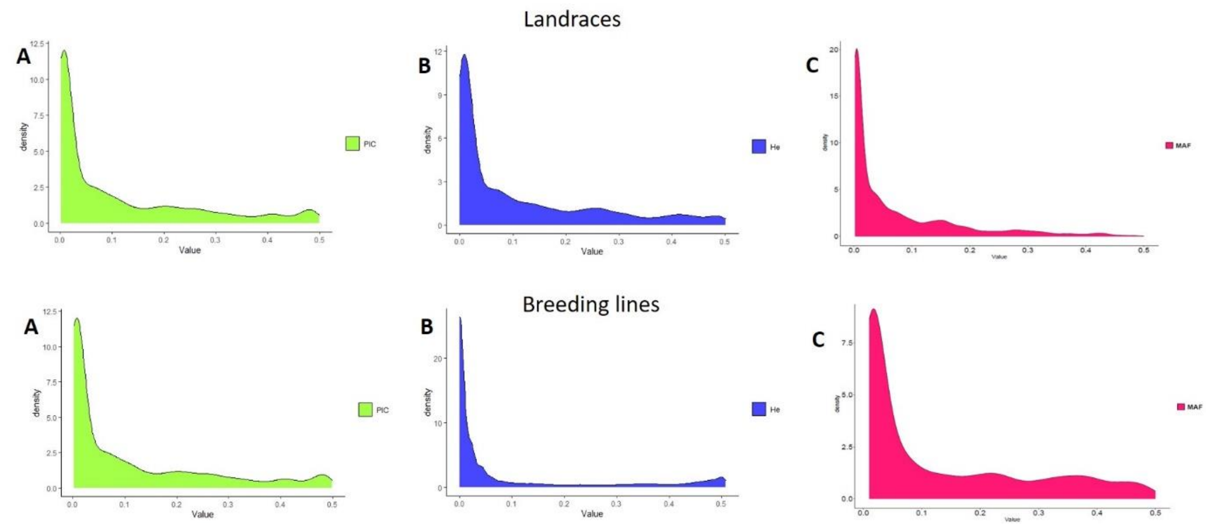

Supplementary Figure 1: Frequency distribution of quality marker parameters among breeding lines and landraces: (A) polymorphism information content (PIC), (B) gene diversity (*He*) and (C) minor allele frequency (MAF) for DArTseq markers in the finger millet diversity panel

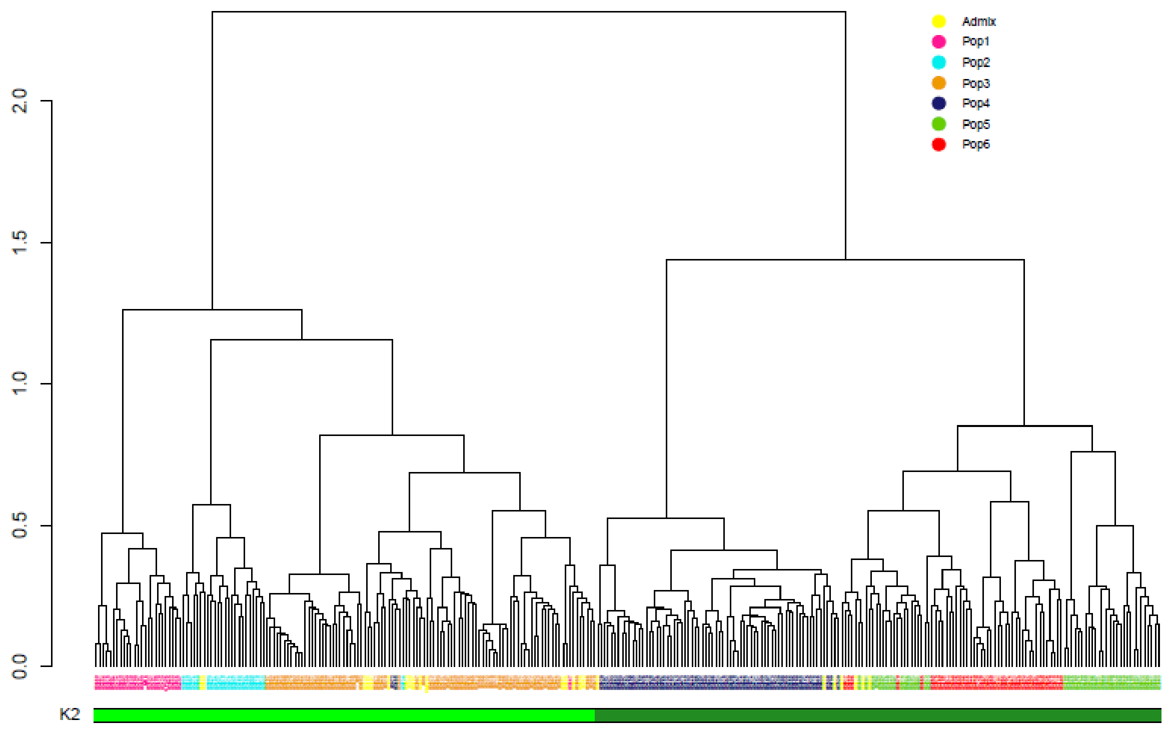

Supplementary Figure 2: Ward.D2 agglomerative hierarchical clustering analysis of finger millet diversity panel based on STRUCTURE results (K6). Note: coloured bars representing major clusters at K2 -- cluster I - Asia (green), cluster II - Africa (dark green); coloured accessions denote the substructure within major clusters: Pop1 and Pop3 represent Indian accessions and Pop2 represents Nepalese accessions, Pop4 represents African highland accessions and Pop5 and 6 represent African lowland accessions.

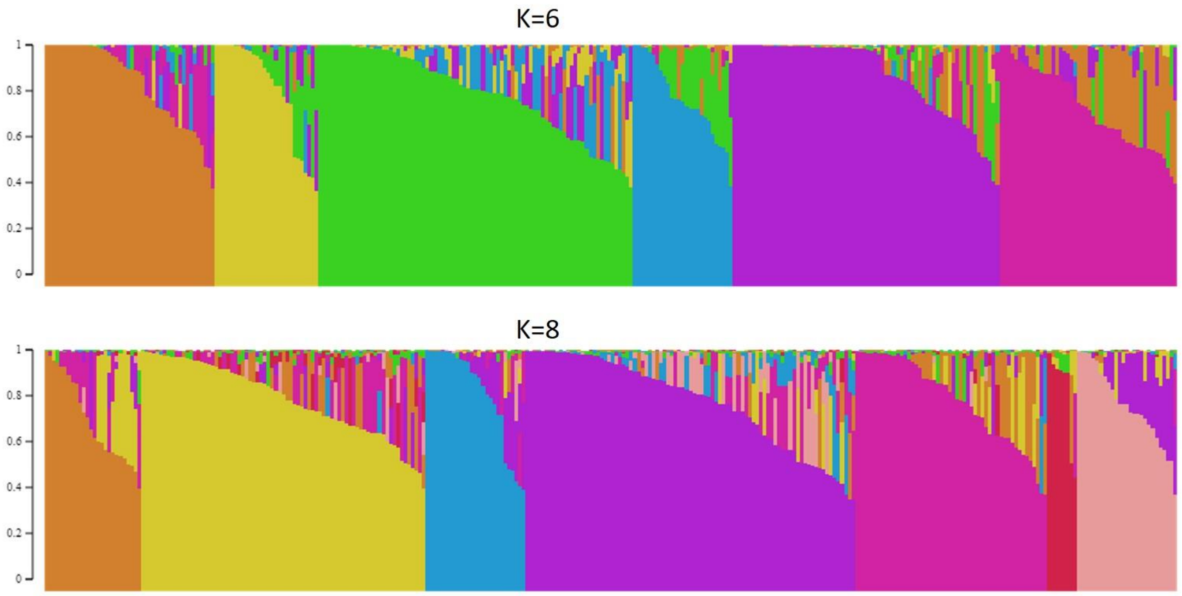

Supplementary Figure 3: Estimated population structure of 306 finger millet accessions on K=6 and K=8
